# First study on the metazoan parasite community of *Crenicichla strigata* (Cichliformes: Cichlidae)

**DOI:** 10.1590/S1984-29612024063

**Published:** 2024-10-07

**Authors:** Leonardo de Oliveira Mota-Júnior, Paulo Venicius Nascimento Santos, David Sales Sousa Valentim, Marcos Sidney Brito Oliveira, Marcos Tavares-Dias

**Affiliations:** 1 Programa de Pós-graduação em Biodiversidade Tropical- PPGBio, Universidade Federal do Amapá – UNIFAP, Macapá, AP, Brasil; 2 Universidade do Estado do Amapá – UEAP, Macapá, AP, Brasil; 3 Embrapa Amapá, Macapá, AP, Brasil

**Keywords:** Amazon, parasitic infection, freshwater fish, dispersion, Amazônia, infecção parasitária, peixe de água doce, dispersão

## Abstract

This study provides the first report of metazoan parasites in *Crenicichla strigata*. From 31 hosts caught in the Jari River basin, in the eastern Amazon region of Brazil, a total of 1454 parasites were collected: *Sciadicleithrum araguariensis*, *Sciadicleithrum joanae*, *Sciadicleithrum satanopercae*, *Posthodiplostomum* sp., *Genarchella genarchella*, *Contracaecum* sp., *Spirocamallanus peraccuratus*, Acarina gen. sp. and *Dolops geayi*. However, the community was dominated by the three species of *Sciadicleithrum* (Monogenea) and there was similar presence of parasites in the larval and adult stages. The total prevalence was 100% and each of the hosts was parasitized by two or three species, which presented random dispersion. Brillouin diversity, parasite species richness, Berger-Parker dominance index and evenness were low. There was positive correlation between the abundance of *Posthodiplostomum* sp. the hosts’ length, while the abundance of *S. peraccuratus* showed negative correlation with the body weight of fish. The abundance of *S. araguariensis, S. joanae* and *S. satanopercae* showed negative correlation with the hosts’ length. The parasite community of *C. strigata* was characterized by low diversity, low richness, low intensity and low abundance of species.

## Resumo

Este estudo descreveu o primeiro relato de parasitos metazoários em *Crenicichla strigata*. De 31 hospedeiros capturados na bacia do Rio Jari, na região da Amazônia oriental brasileira, foi coletado um total de 1.454 parasitos: *Sciadicleithrum araguariensis*, *Sciadicleithrum joanae*, *Sciadicleithrum satanopercae*, *Posthodiplostomum* sp., *Genarchella genarchella*, *Contracaecum* sp., *Spirocamallanus peraccuratus*, Acarina gen. sp. e *Dolops geayi*. Porém, a comunidade foi dominada por essas três espécies de *Sciadicleithrum* (Monogenea) e houve similar presença de parasitos em estágio larval e adulto. A prevalência total foi de100% e os hospedeiros estavam parasitados por duas ou três espécies, que tiveram dispersão randômica. A diversidade de Brillouin, riqueza de espécies de parasitos, índice de dominância de Berger-Parker e equitabilidade foram baixos. Houve correlação positiva da abundância de *Posthodiplostomum* sp. com o comprimento dos hospedeiros, enquanto a abundância de *S. peraccuratus* mostrou correlação negativa com o peso corporal dos peixes. Abundância de *S. araguariensis, S. joane* e *S. satanopercae* apresentou correlação negativa com o comprimento dos hospedeiros. A comunidade parasitária de *C. strigata* foi caracterizada por baixa diversidade, baixa riqueza, baixa intensidade e baixa abundância de espécies.

## Introduction

The Amazon River basin forms part of the major Amazon biome, which presents unequalled aquatic biodiversity. This tropic hydrographic basin of worldwide importance encompasses a complex system of tributaries formed mainly by rivers, channels, lakes, seasonally flooded forest (igapós) and forest creeks (streams), which periodically change their structure and water quality due to sedimentation processes and transportation of dissolved and particulate material, and because of the dry/rainy period seasonality that is characteristic of the Amazon region. Therefore, this hydrographic basin has a large variety of ecosystems with particular characteristics and diverse sizes, in the different South American countries into which the Amazon biome extends (Val, 2019; Abreu et al., 2020). These ecosystems are very dynamic and their constant environmental variations may significantly influence the ichthyofauna and presence of aquatic invertebrates.

Among these tributaries of the Amazon River is the Jari River. Its basin is delimited to the north by Suriname and French Guiana, to the south by the Amazon River and to the west by the state of Pará. The Jari River basin encompasses five municipalities in the state of Amapá (Lucas et al., 2010; Silva & Rauber, 2024). It has dark clear water and is strongly influenced by the daily tides of the Amazon River. Moreover, the urban influence of these municipalities along the Jari River has had significant environmental effects on this basin, with changes to the main bed of the river and sanitary problems due to lack of urban planning. In addition, industrial activities have had an impact, giving rise to changes to the water quality in this hydrographic basin (Lucas et al., 2010; Abreu & Cunha, 2015). These effects may have had an influence on the local biodiversity, which remains little known.

The family Cichlidae Bonaparte, 1840, is formed by freshwater and brackish-water fish, with distribution in Central and South America, along with elsewhere worldwide: Texas, West Indies, Africa, Madagascar, Syria, Israel, Iran, Sri Lanka and the south coast of India (Kullander, 2003; Vanhove et al., 2016; Bisaggio et al., 2023; Froese & Pauly, 2024). This family is recognized to be genus-rich (253), with a high number of species (1786) (Froese & Pauly, 2024). More than 400 species of parasites have been recorded in cichlids worldwide, and around 50% of the parasites have been reported in fish in the Americas (Santacruz et al., 2022). They also have great economic importance (Vanhove et al., 2016) for many regions of the world. Nonetheless, despite the importance of cichlid species and of knowledge of their parasites, which could provide important information on the evolution of parasite-host interactions (Vanhove et al., 2016), the parasite fauna of a large proportion of the cichlid species of the Amazon region remains unknown.

Among the species of cichlids inhabiting the Amazon region, *Crenicichla strigata* Günther, 1862, has been little studies regarding its biology even though it presents wide distribution in many tributaries of the Amazon River basin in Brazil and French Guiana (Santos et al., 2004; Froese & Pauly, 2024). However, this cichlid species, which can reach a length of 30 cm and inhabits the margins of rivers and lakes, has importance as a food source for riverbank human populations of the Amazon region (Santos et al., 2004) and is in demand as an aquarium species, but does not appear on the IUCN list. This is a fish species of carnivorous habits that consumes other smaller fish and aquatic invertebrates, but its reproduction remains unknown (Santos et al., 2004; Froese & Pauly, 2024). Nevertheless, it is known that *C. strigata* presents aggressive behavior: while the females take care of the young offspring, the males defend the territory; and subsequently, both parents guard the offspring when they begin to swim freely (Froese & Pauly, 2024).

Despite the importance and increasing numbers of studies on the communities and infracommunities of parasites of wild fish communities in the Amazon region, no such studies on *C. strigata* are available. However, reports on parasites of other species of cichlids of the genus *Crenicichla* in the Amazon region do exist: these cichlids have been infected by species of Monogenea, Digenea, Nematoda, Cestoda and Crustacea (Table 1). Therefore, the aim of the present study was to provide the first report of the community and infracommunities of metazoan parasites of *C. strigata*.

**Table 1:** Metazoan parasites in fish species of genus *Crenicichla* in Brazil.

**Host species**	**Parasite species**	**Groups**	**Localities**	**References**
*Crenicichla cincta* Regan, 1905	*Ergasilus xinguensis*	Crustacea	Matapi River, state of Amapá	Neves & Tavares-Dias (2019)
*Crenicichla johanna* Heckel, 1840	*Ergasilus xinguensis*	Crustacea	Matapi River, state of Amapá	Neves & Tavares-Dias (2019)
*Crenicichla* sp.	*Dolops geayi*	Crustacea	Janauacá Lake, state of Amazonas	Malta (1982)
*Crenicichla britskii* Kullander, 1982	*Valipora* sp.	Cestoda	Paraná River, state of Paraná	Lehun et al. (2020), Takemoto et al. (2009)
*Crenicichla jaguarensis* Haseman, 1911	Cestoda gen. sp.	Cestoda	Paraná River, state of Paraná	Lehun et al. (2020)
*Crenicichla niederleinii* Holmberg, 1891	*Ascocotyle* sp.	Digenea	Paraná River, state of Paraná	Yamada et al. (2008)
*Crenicichla britskii* Kullander, 1982	Digenea gen. sp.	Digenea	Paraná River, state of Paraná	Lehun et al. (2020)
*Crenicichla britskii* Kullander, 1982	*Austrodiplostomum compactum*	Digenea	Paraná River, state of Paraná	Lehun et al. (2020)
*Crenicichla* sp.	Diplostomidae gen. sp	Digenea	Paraná River, state of Paraná	Lehun et al. (2020)
*Crenicichla* sp.	*Neascus* sp.	Digenea	Paraná River, state of Paraná	Lehun et al. (2020)
*Crenicichla* sp.	*Crassicutis cichlasomae*	Digenea	Paraná River, state of Paraná	Lehun et al. (2020)
*Crenicichla britskii* Kullander, 1982	*Austrodiplostomum compactum*	Digenea	Paraná River, state of Paraná	Machado et al. (2005)
*Crenicichla niederleinii* Holmberg, 1891	*Sciadicleithrum* sp.	Monogenea	Paraná River, state of Paraná	Yamada et al. (2008), Takemoto et al. (2009)
*Crenicichla niederleinii* Holmberg, 1891	Digenea gen sp.	Digenea	Paraná River, state of Paraná	Takemoto et al. (2009)
*Crenicichla britskii* Kullander, 1982	*Sciadicleithrum joanae*	Monogenea	Paraná River, state of Paraná	Lehun et al. (2020)
*Crenicichla* sp.	Monogenea gen. sp.	Monogenea	Paraná River, state of Paraná	Lehun et al. (2020)
*Crenicichla niederleinii* (Holmberg, 1891)	*Sciadicleithrum satanopercae*	Monogenea	Paraná River, state of Paraná	Yamada et al. (2009)
*Crenicichla britskii* Kullander, 1982	*Sciadicleithrum satanopercae*	Monogenea	Paraná River, state of Paraná	Yamada et al. (2009)
*Crenicichla britskii* Kullander, 1982	Nematoda gen. sp.	Nematoda	Paraná River, state of Paraná	Lehun et al. (2020), Takemoto et al. (2009)
*Crenicichla* sp.	*Contracaecum* sp.	Nematoda	Paraná River, state of Paraná	Lehun et al. (2020)
*Crenicichla* sp.	*Hysterothylacium* sp.	Nematoda	Paraná River, state of Paraná	Lehun et al. (2020)
*Crenicichla* sp.	*Procamallanus peraccuratus*	Nematoda	Paraná River, state of Paraná	Lehun et al. (2020)
*Crenicichla* sp.	*Procamallanus peraccuratus*	Nematoda	Crôa River, Santa Rosa River, Juruá River, Gama River and Môa River, state of Acre	Virgilio et al. (2022)
*Crenicichla jaguarensis* Haseman, 1911	*Spirocamallanus inopinatus*	Nematoda	Paraná River, state of Paraná	Lehun et al. (2020)
*Crenicichla* sp.	*Spirocamallanus inopinatus*	Nematoda	Crôa River, Santa Rosa River, Juruá River, Gama River and Môa River, state of Acre	Virgilio et al. (2022)
*Crenicichla* sp.	*Heliconema izecksohni*	Nematoda	Crôa River, Juruá River, Paranã River and Môa River, state of Acre	Virgilio et al. (2022)
*Crenicichla haroldoi* Luengo & Britski, 1974	*Spirocamallanus peraccuratus*	Nematoda	Paraná River, state of Paraná	Moravec et al. (1993)

## Material and Methods

**Figure 1:**
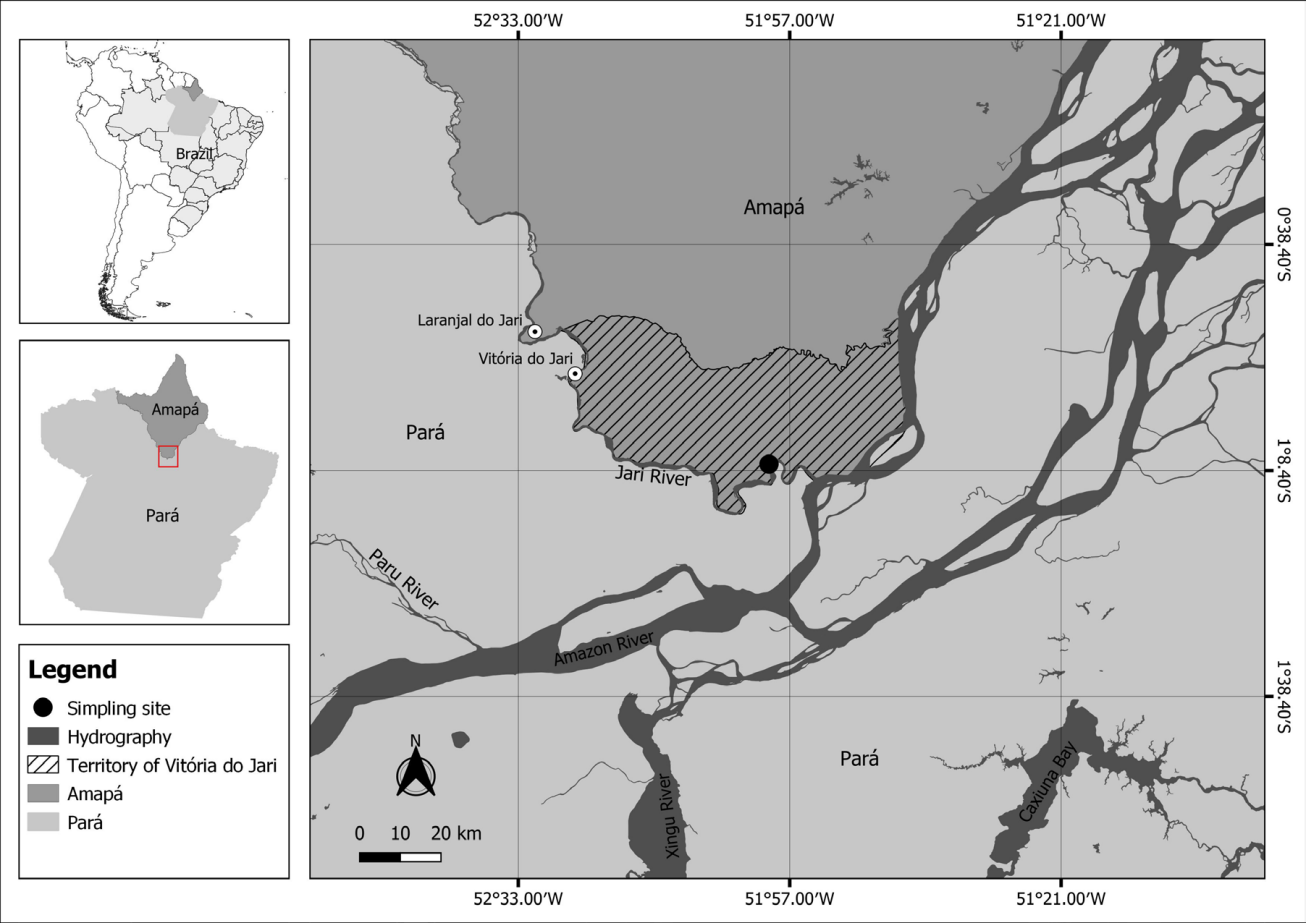
Sampling site of *Crenicichla strigata* in Jari River basin, region from the eastern Brazilian Amazon.

Thirty-one specimens of *C. strigata* were collected from the Jari River basin, in the Jarilândia district, municipality of Vitória do Jari, in state of Amapá, Brazil (Figure 1). This fish were caught using gillnets of mesh size 25 and 30 mm between knots. Each fish was weighed (g) and measured along its standard length (cm). All the fish then underwent necropsy for parasitological analyses. Their mouth, gills, operculum and fins were examined to detect any presence of ectoparasites. The gastrointestinal tract and viscera were removed and examined for presence of endoparasites using a stereomicroscopy. The collection, fixing, conservation and preparation of parasites for identification were done in accordance with previous recommendations (Eiras et al., 2006; Thatcher, 2006). The ecological terms used (prevalence, mean intensity and mean abundance) were as recommended by Bush et al. (1997). The following were determined: Brillouin diversity index (*HB*), uniformity (evenness) (*E*), Berger-Parker dominance index (*d*), parasite species richness (Magurran, 2004) and dominance frequency, i.e. the percentage of the infracommunities in which a given parasite species is numerically dominant (Rohde et al., 1995; Magurran, 2004). The diversity indices were evaluated using the Diversity software (Pisces Conservation Ltd, UK). The dispersion index (DI) and Poulin discrepancy index (D) were calculated using the Quantitative Parasitology 3.0 software, to detect the distribution pattern of the parasite infracommunities (Rózsa et al., 2000), for species with prevalence > 10%. The significance of the DI, for each infracommunity, was calculated using the *d-*statistical test (Ludwig & Reynolds, 1988). Spearman’s correlation coefficient (*rs*) was used to determine possible correlations between parasite abundance and the hosts’ length, body weight, Brillouin diversity index and parasite species richness (Zar, 2010). The parasites were identified in accordance with the recommendations of Thatcher (2006), Moravec (1998) and Kohn et al. (2007).

## Results

The fish examined had a mean weight of 49.4 ± 9.8 g (28.6 to 72.0) and mean length of 15.8 ± 1.1 cm (13.4 to 18.2). Among them, a total of 1454 metazoan parasites were collected. These included the following: *Sciadicleithrum araguariensis* Paschoal, Scholz, Tavares-Dias & Luque; *Sciadicleithrum joanae* Yamada, Takemoto, Bellay & Pavanelli, 2009; *Sciadicleithrum satanopercae* Yamada, Takemoto, Bellay & Pavanelli, 2009 (Dactylogyridae); larvae of *Posthodiplostomum* Dubois, 1936 (Diplostomidae); *Genarchella genarchella* Travassos, Artigas & Pereira, 1928 (Derogenidae); larvae of *Contracaecum* (Anisakidae); *Spirocamallanus peraccuratus* Pinto, Fábio & Rolas, 1976 (Camallanidae); Acarina gen. sp.; and *Dolops geayi* Bouvier, 1897 (Argulidae) (Voucher: 178P-186P - Instituto de Pesquisas Científicas e Tecnológicas do Amapá/IEPA, Brazil). However, the parasite community was dominated by the three species of *Sciadicleithrum* (Monogenea) on the hosts’ gills (Table 2).

**Table 2:** Metazoan parasites of *Crenicichla strigata* (N = 31) from the Jari River basin, in the eastern Brazilian Amazon.

**Parasite species**	**P (%)**	**MI**	**MA ± SD**	**FD (%)**	**TNP**	**SI**
*Sciadicleithrum araguariensis*, *Sciadicleithrum joanae* and *Sciadicleithrum satanopercae*	100	33.5	33.5 ± 14.8	71.3	1037	Gills
*Posthodiplostomum* sp. (metacercarie)	48.4	8.1	3.9 ± 6.6	8.3	121	Mesentery
*Posthodiplostomum* sp. (metacercarie)	19.4	4.0	0.8 ± 2.1	1.7	24	Swim bladder
*Posthodiplostomum* sp. (metacercariae)	6.5	1.0	0.1 ± 0.4	0.2	3	Intestine
*Posthodiplostomum* sp. (metacercariae)	25.8	3.0	0.8 ± 1.5	1.7	24	Liver
*Genarchella genarchella*	6.5	1.5	0.1 ± 0.4	0.1	3	Swim bladder
*Genarchella genarchella*	6.5	1.0	0.06 ± 0.2	0.1	2	Intestine
*Contracaecum* sp. (larvae)	83.9	2.6	2.2 ± 1.5	4.7	68	Mesentery
*Contracaecum* sp. (larvae)	9.7	1.0	0.1 ± 0.3	0.2	3	Intestine
*Contracaecum* sp. (larvae)	6.5	1.0	0.06 ± 0.2	1.0	2	Abdominal cavity
*Spirocamallanus peraccuratus*	9.7	1.3	0.1 ± 0.4	0.3	4	Mesentery
*Spirocamallanus peraccuratus*	77.4	3.5	2.7 ± 2.5	5.8	85	Intestine
Acarina gen. sp.	35.5	1.9	0.7 ± 1.2	1.4	21	Gills
*Dolops geayi*	6.5	1.0	0.06 ± 0.2	0.1	2	Gills

P: Prevalence; MI: Mean intensity; MA: Mean abundance; FD: Frequency of dominance; TNP: Total number of parasites; SI: Site of infection; SD: Standard deviation.

The parasites of *C. strigata* presented random a low dispersion (Table 3). Among the fish analyzed, 100% were infected by one or more species of parasites. However, there was similar presence of ectoparasites and endoparasites in these hosts and similar presence of parasites in larval and adult stages (Table 4). The Brillouin diversity index ranged from 0.25 to 1.5, parasite richness from 2 to 4, evenness from 0.2 to 0.7 and Berger-Parker dominance index from 0.5 to 0.9. (Table 5).

**Table 3:** Dispersion index (DI), *d*-statistic (*d*) and discrepancy index (D) for the metazoan parasite infracommunities of *Crenicichla strigata* (N=31) from the Jari River basin, in the eastern Brazilian Amazon.

**Species of parasites**	**DI**	**D**	***d***	**Type of dispersion**
*Posthodiplostomum* sp.	1.02	0.319	0.142	Random
*Contracaecum* sp.	0.95	0.339	-0.311	Random
*Spirocamallanus peraccuratus*	1.27	0.405	1.048	Random
*Sciadicleithrum araguariensis, Sciadicleithrum joane* and *Sciadicleithrum satanopercae*	1.52	0.276	1.869	Random

**Table 4:** Component community of metazoan parasites in *Crenicichla strigata* (N=31) from the Jari River basin, in the eastern Brazilian Amazon.

**Characteristics**	**Values**
Number of examined hosts	31
Prevalence (%) of parasites	100%
Total number of parasites	1454
Species of ectoparasites	5
Ectoparasites percentage	55.5
Ectoparasite (larvae) species	2
Species of endoparasites	4
Endoparasites percentage	44.5
Endoparasite (adults) species	2
Endoparasite (larvae) species	2

**Table 5:** Diversity descriptors for the metazoan parasite community of *Crenicichla strigata* from the Jari River basin, in the eastern Brazilian Amazon.

**Diversity indices**	**Mean ± SD**	**Ranges**
Species richness	3.4 ± 0.6	2-4
Brillouin diversity index (*HB*)	0.6 ± 0.2	0.25-1.5
Berger-Parker dominance index	0.7 ± 0.1	0.5-0.9
Evenness (*E*)	3.4 ± 0.6	0.2-0.7

The Brillouin diversity did not showed any significant correlation with host weight (*rs* = -0.21, p = 0.24) or with host body length (*rs* =-0.04, p = 0.82). The parasite species richness did not present any significant correlation with weight (*rs* = -0.02, p = 0.89) or body length (*rs* = -0.11, p = 0.53) among the fish examined.

The abundance of *Posthodiplostomum* sp. showed positive correlation (*rs* = 0.38, p = 0.03) with the hosts’ length, but no significant correlation with their weight (*rs* = -0.009, p = 0.95). The abundance of *Contracaecum* sp. did not show any correlation (*rs* = 0.20, p = 0.26) with the length and weight of the hosts (*rs* = 0.34, p = 0.50). The abundance of *S. peraccuratus* did not show any significant correlation (*rs* = -0.12, p = 0.50) with the hosts’ length, but showed a negative correlation with their body weight (*rs* = -0.44, p = 0.01). The abundance of *S. araguariensis, S. joanae* and *S. satanopercae* showed negative correlation (*rs* = -0.36, p = 0.04) with the hosts’ length, but no significant correlation with their body weight (*rs* = -0.08, p = 0.64).

**Figure 2:**
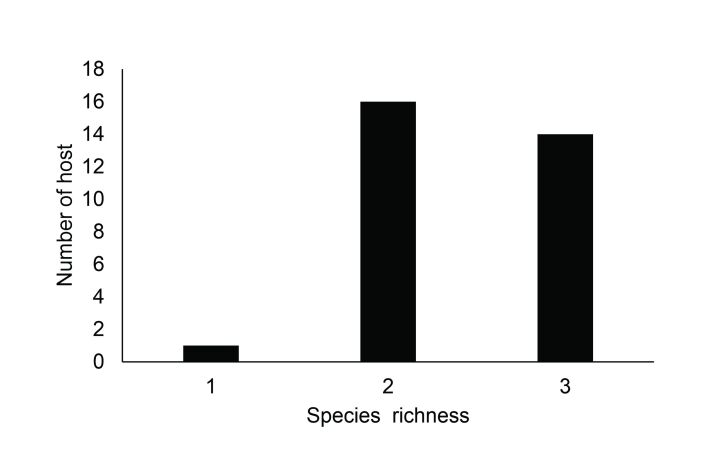
Species richness of metazoan parasites in *Crenicichla strigata* from the Jari River basin, in the eastern Brazilian Amazon.

There was a predominance of hosts parasitized by two or three species (Figure 2).

## Discussion

The parasite community of *C. strigata*, in the Jari River basin, was formed by three species of Monogenea, two of Digenea, two of Nematoda, one of Acarina gen. sp. and one of Crustacea. These parasites were *Sciadicleithrum araguariensis*, *S. joanae*, *S*. *satanopercae*, *Posthodiplostomum* sp., *G. genarchella*, *Contracaecum* sp., *Spirocamallanus peraccuratus*, mites and *D*. *geayi*. All of them were the first records for *C. strigata*. The parasite community of *C. strigata* was dominated by the three species of *Sciadicleithrum* (Monogenea). However, previous studies showed that *S. joanae*, *S*. *satanopercae*, larvae of *Contracaecum* sp. and *Spirocamallanus peraccuratus* have already been reported from other species of the genus *Crenicichla* in Brazil (Table 1). In addition, we found similar occurrence of endoparasites and ectoparasites in *C. strigata*, and of parasites in adult and larval forms.

In *C. strigata*, there was similar presence of ectoparasites and endoparasites, well as similar presence of metazoan parasites in larval and adult stages. In contrast, in *Geophagus brasiliensis* Quoy & Gaimard, 1824, from reservoirs in the state of Paraná, ectoparasites were more frequent. This may have been because the conditions were favorable for transmission of *Sciadicleithrum frequens* Bellay, Takemoto, Yamada & Pavanelli 2008 and other species of monogeneans Dactylogyridae that were not identified. These are ectoparasites that do not need intermediate hosts (Bellay et al., 2012). However, in *Gymnogeophagus balzanii* Perugia, 1891, from the Pantanal region of the state of Mato Grosso do Sul, 70% of the metazoan parasites were in larval stage and only 30% in the adult stage (Bisaggio et al., 2023). These different results can be attributed to differences between species and between their hosts’ feeding habits, along with the different environment within which these cichlid species were caught, in addition other factors. Environmental features may influence parasitic communities, and this influence tends to be more intense, especially on ectoparasites that are in direct contact with the external environment (Bisaggio et al., 2023). Variation in these patterns for metazoan parasites may also be related to the abundance of intermediate hosts in environment, which are eaten by the fish (Neves et al., 2013). Furthermore, host fish species at higher levels in the trophic web would be exposed to greater numbers of endohelminths larvae from a broader range of parasite taxa through their diet than would those at lower trophic levels over evolutionary time (Baia et al., 2018).

In *C. strigata,* random distribution pattern was found with regard to *Posthodiplostomum* sp., *Contracaecum* sp., *S. araguariensis*, *S. joanae*, *S*. *satanopercae* and *Spirocamallanus peraccuratus*. In contrast, uniform dispersion was reported in relation to *Amplexibranchius bryconis* Thatcher & Paredes, 1985, in *Mylossoma aureum* Spix & Agassiz, 1829, and *Mylossoma duriventre* Cuvier, 1818, from the Madeira River, in the Brazilian Amazon region. However, this dispersion pattern is only rarely observed among parasites of wild populations (Pelegrini et al., 2024). A random dispersion pattern was also reported in relation to *Anacanthorus daulometrus* Cohen, Kohn & Boeger 2012 and *Lernaea cyprinacea* Linnaeus, 1758, in *Salminus brasiliensis* Cuvier, 1816, and *Brycon orbignyanus* Valenciennes, 1850, from the Paraná River, Argentina (Furlan et al., 2023). On the other hand, aggregated dispersion pattern is more common for freshwater fish, for a variety of reasons (Bisaggio et al., 2023; Pelegrini et al., 2024). This pattern enables greater contact between parasite specimens, thus favoring their reproduction and facilitating coexistence between different species in a single population of host fish (Pelegrini et al., 2024). In wild populations of fish with high numbers of infectious phases and differentiated immunological responses, increased levels of parasite aggregation may occur. In such situations, the small numbers of hosts with higher levels of infection would be more likely to die, thus resulting in random dispersion of parasites (Vidal-Martínez et al., 2014).

In *C. strigata*, the parasite community presented dominance by *S. araguariensis*, *S. joanae* and *S. satanopercae*, as also indicated by the Berger-Parker dominance index. Moreover, there was no correlation between the abundances of these ectoparasites and the Brillouin diversity. The Brillouin diversity in *C. strigata* was lower than what was reported for *M. aureum* and *M. duriventre*, but the parasite species richness was similar (Pelegrini et al., 2024). In contrast, the diversity index for *C. strigata* was greater than what was reported for *G. balzanii*, while the parasite species richness was lower (Bisaggio et al., 2023). However, Pelegrini et al. (2024) stated that for more precise conclusions to be reached regarding comparisons of diversity indices between different populations of fish, the respective sampling areas of the hosts and their equivalences would need to be considered. In other words, data rarefaction curves for the parasites collected should be used in order to make better comparative analyses.

In wild fish populations, levels of infections by monogeneas has been associated with various environmental factors. For example, high levels of infection by *Gussevia asota* Kritsky, Thatcher & Boeger, 1989, *Gussevia astronoti* Kritsky, Thatcher & Boeger, 1989, and *Gussevia rogersi* Kritsky, Thatcher & Boeger, 1989, has been reported in *Astronotus ocellatus* Agassiz, 1831 from Pracuúba Lake, state of Amapá, due to seasonal variation in water quality (Neves et al., 2013). High infection by *Anacanthorus cladophallus* Van Every & Kritsky, 1992, *Anacanthorus paraspathulatus* Kritsky, Boeger & Van Every, 1992, and *Mymarothecium* spp. in *M. aureum* and *M. duriventre* from the Madeira River was also reported (Pelegrini et al., 2024). Monogeneans *Sciadicleithrum kritskyi* Bellay, Takemoto, Yamada & Pavanelli 2009; *Sciadicleithrum paranaensis* Bellay, Takemoto, Yamada & Pavanelli 2009 and *Sciadicleithrum geophagi* Kritsky, Thatcher & Boeger, 1989 were also predominated in *Geophagus proximus* Castelnau, 1855) due to high contact with the infecting forms (oncomiracidium) of these monoxenic ectoparasites (Oliveira et al., 2017). In general, for most species of monogeneans, high levels of parasitism can occur in wild host fish in lentic environments and/or those with low water quality, given that the lifecycle is direct, thus facilitating parasite reproduction (Koskivaara & Valtonen, 1992; Neves et al., 2013). Therefore, high levels of infection by different species of monogeneans can occur in different species of host fish in distinct natural environments.

Species of the genus *Posthodiplostomum* are distributed in all parts of the world (Ritossa et al., 2013). In general, the transmission of species of digeneans is related to the life habits of the host fish species and their position in the trophic web (Santacruz et al., 2022). In *C. strigata*, larvae of *Posthodiplostomum* sp. presented moderate levels of infection. This digenean has a lifecycle involving two intermediate hosts: one species of mollusk (primary) and one species of fish (secondary), while fish-eating birds are the definitive hosts (Ritossa et al., 2013). Thus, *C. strigata* is an intermediate host for larvae of *Posthodiplostomum* sp. Among the hosts in the Jari River basin, there was greater prevalence and abundance of *Posthodiplostomum* sp. than of *G*. *genarchella*, but *C. strigata* is a definitive host for the latter species of digenean. However, both of these digeneans are parasites without specificity for hosts, given that they have been reported infecting different freshwater fish species in Brazil (Kohn et al., 2007; Neves et al., 2013; Tavares-Dias et al., 2017).

In the mesentery of *C. strigata*, larvae of *Contracaecum* sp. were found at high prevalence and low levels of intensity and abundance. However, these infection high rates were not found in the intestine and abdominal cavity of this host. This nematode has a phase of life in species of copepods, which form primary intermediate hosts, and another in fish, which form secondary intermediate or paratenic hosts, while adults of this species are found in the proventriculus/ventriculus of fish-eating birds, which form the definitive hosts (Pinheiro et al., 2019; Moraes et al., 2024; Pelegrini et al., 2024). Larvae of *Contracaecum* sp. are nematodes without specificity of hosts, given that they occur in a variety of fish species, and at high infection rates in some of them (Pinheiro et al., 2019; Pelegrini et al., 2024). Furthermore, larvae of *Contracaecum* sp. are Anisakidae of great importance for public health, due to their zoonotic potential for humans (Pinheiro et al., 2019; Moraes et al., 2024; Pelegrini et al., 2024).

*Spirocamallanus peraccuratus* presented low rates of infection in the intestine of *C. strigata*, an infection pattern that was not observed in the mesentery of this host. However, low levels of infection by *S*. *peraccuratus* also occurred in the intestine of *G. brasiliensis* from rivers in the state of Paraná, Brazil (Bellay et al., 2012). This species of camallanid was also reported infecting the intestine of a species of the genus *Crenicichla* from the Paraná River basin (Table 1) and the intestine of the cichlid *Biotodoma cupido* Heckel, 1840 from five different rivers in the Brazilian western Amazon region (Virgilio et al., 2022). Moravec (1998) reported that the nematode *S. peraccuratus* has distribution in Brazil and Paraguay, mainly parasitizing cichlids, and that although its lifecycle remains unknown, fish are probably the definitive hosts.

Crustaceans are one of the main groups of the phylum Arthropoda and a significant proportion of their species are parasites, which therefore can infect a variety of fish species. Low levels of infection by *D. geayi* were observed in *C*. *strigata* from the Jari River basin. Similar results were reported in relation to *Cichlasoma amazonarum* Kullander, 1983, cultivated in Peru (Lo et al., 2011), and *Colossoma macropomum* Cuvier, 1818, in the state of Amazonas (Brazil), and also in relation to *Hoplias malabaricus* Bloch, 1794, and *A. ocellatus* from Janauacá Lake, in the state of Amazonas (Malta, 1982); *Crenicichla* sp. (Table 1); and *S. brasiliensis* and *B. orbignyanus* from the Paraná River, Argentina (Furlan et al., 2023). In Brazil, *D. geayi* has distribution in the basins of the Paraná and Amazon Rivers (Tavares-Dias et al., 2015), but it has also been reported from Venezuela, French Guiana, Bolivia and Paraguay (Suárez-Morales, 2020). In wild fish populations, infection by *D. geayi* are generally low and rarely cause signs of pathological conditions in their hosts (Lo et al., 2011), as was observed in the present study.

The behavior of fish does not contribute to infections caused by mites. Moreover, there is controversy regarding parasitism by mites in fish, given that some studies have considered them to be parasites, while others have disagreed about this. In *Oreochromis niloticus* Linnaeus, 1758, species of mites were identified on the skin, fins, gills and mouth of their hosts. Although fish do not form part of the habitual lifecycle of mites, it can be presumed that high proliferation of mites may have negative effects on the health of infected fish (Olmeda et al., 2011). However, on the gills of *C*. *strigata*, the abundance and intensity of mites were low, similar to what was observed in relation to *G. brasiliensis* (Bellay et al., 2012). Lehun et al. (2020) also found the presence of mites on the gills and body surface of *Geophagus sveni* Lucinda, Lucena & Assis, 2010, and *Serrasalmus marginatus* Valenciennes, 1837, from the floodplain of the upper Paraná River, in Brazil.

The body size of host fish, which is a factor related to their age, has been considered to be a determinant for parasite abundance that partly explains the variation of some parasite infracommunities (Poulin & Justine, 2008; Bellay et al., 2012; Pelegrini et al., 2024). In general, larger fish provide more space in which to host more species of parasites (Pelegrini et al., 2024). In *C*. *strigata*, there was positive correlation between the abundance of *Posthodiplostomum* sp. with the hosts’ length. The abundance of *S. peraccuratus* showed a negative correlation with the weight of the fish examined, and the abundance of *S. araguariensis, S. joanae* and *S. satanopercae* presented negative correlation with body length of hosts. However, the small variation in body size among the specimens of *C. strigata* that were collected, particularly regarding their length, was influenced by the nets used to catch them (net sizes of 25 and 30 mm between knots). This therefore affected the correlations between parasite abundance and the size of the hosts examined. Nonetheless, variations in body size alone cannot explain differences in abundance between parasite species with regard to host body size, given that abundance is also related to the parasite species’ life history characteristics, which sustain their reproductive rates and population growth (Poulin & Justine, 2008).

This first study on the community of metazoan parasites of *C. strigata* was characterized by low diversity, low species richness, low intensity and low abundance for most of the parasite species recovered. This fish forms both an intermediate and a definitive host for parasites in the Jari River basin. The size of the hosts had little influence on the parasite community, but the carnivorous diet of these fish was an important factor structuring this community. Lastly, because of the possibly increasing anthropic interference in the water quality of the Jari River basin, caused by industrial and urban development, future studies should be conducted with the aim of understanding and evaluating the extent of these impacts on the parasite community of this fish of the Amazon basin.
